# Cutoff point of TyG index for metabolic syndrome in Brazilian farmers

**DOI:** 10.20945/2359-3997000000401

**Published:** 2021-09-29

**Authors:** Júlia Rabelo Santos Ferreira, Eliana Zandonade, Olívia Maria de Paula Alves Bezerra, Luciane Bresciani Salaroli

**Affiliations:** 1 Universidade Federal do Espírito Santo Programa de Pós-Graduação em Saúde Coletiva (PPGSC) Vitória ES Brasil Programa de Pós-Graduação em Saúde Coletiva (PPGSC), Universidade Federal do Espírito Santo (UFES), Vitória, ES, Brasil; 2 Universidade Federal de Ouro Preto Escola de Nutrição Departamento de Nutrição Clínica e Social Ouro Preto MG Brasil Departamento de Nutrição Clínica e Social, Escola de Nutrição, Universidade Federal de Ouro Preto (UFOP), Ouro Preto, MG, Brasil; 3 Universidade Federal do Espírito Santo Programa de Pós-Graduação em Nutrição e Saúde (PPGNS) Vitória ES Brasil Programa de Pós-Graduação em Nutrição e Saúde (PPGNS), Universidade Federal do Espírito Santo (UFES), Vitória, ES, Brasil

**Keywords:** Metabolic syndrome, insulin resistance, TyG index

## Abstract

**Objective:**

Thus, the aim of this study was to identify the cutoff point of the TyG index for the diagnosis of insulin resistance (IR), according to two different diagnostic criteria of metabolic syndrome in a rural Brazilian population.

**Materials and methods:**

The study population consisted of 790 family farmers from 18 to 59 years old. The triglyceride-glucose index (TyG index) was calculated, and the Metabolic Syndrome was defined using the NCEP-ATPIII and IDF criteria. Mann-Whitney U test was used to analyze the association of quantitative and qualitative variables. When the qualitative variable had three or more categories, the comparison between the means was performed by the Kruskal-Wallis test (using the Mann-Whitney U Test two by two to identify the differences). For correlations, Spearman’s correlation test was used. The cutoff values of TyG index for MetS were obtained using the Receiver Operating Characteristic (ROC) curve analysis with the area under the curve (AUC) and the Youden Index.

**Results:**

The median TyG values increased according to the aggregation of the components of MetS. The AUCs and Youden’s cutoff point for TyG index according to the NCEP and IDF diagnostic criteria were 0.873, Ln 4.52 (sensitivity: 84.30%; specificity: 75.75%), and 0.867, Ln 4.55 (sensitivity: 80.0%; specificity: 79.82%), respectively.

**Conclusion:**

A cutoff point of Ln 4.52 was defined, and it can be used both in clinical practice and epidemiological studies. It represents an important tool for promotion, protection and recovery health of rural populations.

## INTRODUCTION

Metabolic syndrome (MetS) is a multifactorial and highly prevalent condition, characterized by a set of abnormalities that include abdominal obesity, hypertension, dyslipidemia and elevated blood glucose. From its first description and the studies carried out by Reaven ( 1 ), it was demonstrated that the link between those metabolic and hemodynamic abnormalities in the same individual was due to insulin resistance (IR). Individuals with IR are at significant risk of developing cardiovascular diseases (CVD) ( 2 ), lipid abnormalities, elevated blood pressure and glucose intolerance ( 1 ). IR is a state of peripheral tissue insensitivity to the effects of insulin ( 3 ). This condition has been linked to obesity, diabetes mellitus type 2 (DM2) and recent studies have also found that IR plays a key role in the development of other chronic diseases, such as hypertension, cancer, polycystic ovary syndrome, chronic kidney disease and brain disorders ( 4 ).

The scientific literature still lacks studies determining the prevalence of IR in the Brazilian population, but a study evaluating IR in a representative sample of Brazilian workers found a prevalence of 10.4% ( 5 ). In rural populations, this is even more scarce. Until then, a study found a prevalence of 24.2% of IR in two rural communities in the state of Minas Gerais ( 6 ).

The gold standard method for the diagnosis of IR is the hyperinsulinemic euglycemic clamp (HIEC) ( 7 ), a measure of peripheral glucose uptake in situations of high insulin concentrations. However, this method is complex, invasive and expensive, and is not incorporated into the clinical routine, especially in public health. Other substitute methods were created, such as the Homeostases Model Assessment-Insulin Resistance (HOMA-IR) index, calculated based on fasting glucose and insulin levels ( 8 ), which also has limitations, including the determination of insulin.

In this scenario, the need to develop an insulin-free substitute index was realized to assess IR. Studies have shown that an increase in serum triglyceride (TG) can compromise muscle glucose metabolism and lead to a decrease in insulin sensitivity ( 9 ). Thus, the triglyceride-glucose index (TyG index) ( 3 ) was proposed, which demonstrated high sensitivity and specificity when compared to the HIEC in the IR assessment ( 10 ), and even superiority in relation to HOMA-IR ( 11 ).

IR is one of the main underlying mechanisms of MetS and for this reason, several population studies have used MetS as an IR indicator and provided cutoff values for HOMA-IR using Receiver Operating Characteristic (ROC) curves for MetS ( 12 - 14 ). However, studies using the TyG index for this purpose are still scarce, especially in adult rural populations.

Considering that TyG index is easy to apply and has been associated with several pathologies, such as cardiovascular events ( 15 ), arterial hypertension ( 16 , 17 ) and diabetes mellitus ( 18 , 19 ), it could act as an adjunct to risk screening in individuals with little access to health services. In this scenario, the rural population and agricultural workers stand out, who, in addition to being exposed to risks common to the entire population (age group, gender, stress, violence, dietary and behavioral factors, such as physical inactivity, smoking, alcoholism), are also exposed to risks and damages inherent to working in the field, such as accidents with hand tools and machines, accidents with poisonous animals, exposure to endemic parasitic agents, repetitive trauma disorders, exposure to pesticides, respiratory diseases due to exposure to stored grain particles, mites, pollen, among others ( 20 ). In addition, some authors have demonstrated that rural populations had a high prevalence of cardiovascular risk factors ( 21 , 22 ), causing this category of workers to get sick and die in a very particular way, caused by this double exposure ( 23 ).

To date, there are no studies elucidating a cutoff point for TyG index based on MetS in Brazil. The objective of this article was to identify the cutoff point of the TyG index for the diagnosis of IR, according to two different diagnostic criteria of MetS in family farmers in a city in Espírito Santo, Brazil.

## MATERIALS AND METHODS

### Study design and population

Cross-sectional analytical observational epidemiological study conducted in the municipality of Santa Maria de Jetibá, mountain region of Espírito Santo, Brazil. Family farmers registered in the Family Health Program, aged between 18 and 59 years, not pregnant, whose main source of income is agriculture and were working actively for at least 6 months were included.

To calculate the sample size, the Epidat program version 3.0 was used, considering a prevalence of 20% ( 24 ), sensitivity and specificity at 80%, precision of 6.5%, significance level of 5% and power analysis of 80%. The minimum sample size is 731 people. Predicting possible losses, 10% were added, reaching a total of 806 people. The participants were selected based on a stratified sampling, considering the number of families by community health agents and respecting the proportionality between the health regions of the municipality. In families with more than one eligible individual, only one individual was included, to avoid the interdependence of information. In case a participant refuses to participate or is absent during data collection, a new participant on the waiting list was called, respecting the sex and region of origin of the dropout. The final sample consisted of 790 individuals (loss of 1.98%).

### Data collection

The data were collected between the months of December 2016 and April 2017 by a team of trained researchers. The variables of the present study included sociodemographic, biochemical and clinical data and waist circumference (WC) measures.

Sociodemographic data were obtained through the application of a semi-structured questionnaire, and the variables included sex (male; female), age (decades), marital status (single; married or living in a stable relationship; separated, divorced or widowed), schooling (less than 4 years of study; 4 to 8; more than 8), race/color self-referenced (whites; nonwhites) and socioeconomic class, according to Brazil Economic Classification Criterion (ABEP) ( 25 ).

The WC was measured with a Sanny inelastic measuring tape (model TR-4010^®^) positioned between the upper border of the iliac crest and the last rib ( 26 ). Three non-consecutive measurements were made, the first being discarded and the average of the last two considered as the final measurement.

Biochemical data were obtained by collecting blood after 12 hours of fasting. The determination of total cholesterol was carried out using the colorimetric enzymatic method with the Cholesterol Liquicolor^®^ Kit (InVitro Diagnóstica Ltda.). For HDL cholesterol, the method used was the colorimetric enzyme with the Kit HDL Cholesterol precipitation^®^ (InVitro Diagnóstica Ltda.) and the determination of LDL cholesterol was performed using the Friedewald formula ( 27 ). Triglycerides were determined using the colorimetric enzymatic method with the Kit Triglycerides Liquicolor mono^®^ (InVitro Diagnóstica Ltda). Blood glucose was determined by the colorimetric enzymatic method with the Enzymatic Glucose Kit^®^ (InVitro Diagnóstica Ltda.).

Blood pressure measurements were performed according to the procedures described in the protocol of the VII Brazilian Hypertension Guideline ( 28 ), using an Omron Automatic HEM – 7200^®^ pressure monitor, properly calibrated and validated by Inmetro (National Institute of Metrology, Quality and Technology). The measurements were performed during the interview, with four repetitions per individual. The first measurement was discarded and the fourth measurement was only used if the difference between the second and third measurements was greater than 4 mmHg.

The TyG index was calculated from the equation: Ln [fasting triglycerides (mg/dL) x fasting glycemia (mg/dL)]/2, and expressed on a logarithmic scale ( 3 ). MetS was defined using the NCEP-ATPIII criteria ( 29 ) (presence of at least three of the following criteria: WC > 88 cm for women or > 102 cm for men, HDL < 50 mg/dL for women or < 40 mg/dL for men, TG ≥ 150 mg/dL, blood pressure with cutoff values considering 130/85 mmHg and fasting glucose ≥ 110 mg/dL) and IDF ( 30 ) (reduces borderline values for fasting blood glucose ≥ 100 mg/dL and for WC > 80 cm for women and > 90 cm for men; having to present WC above the established limit and two more criteria to be classified as MetS). The use of antihypertensive and/or antidiabetic medication are considered as criteria for MetS, since it classifies the individual as hypertensive and/or diabetic, respectively.

### Statistical analyzes

Statistical analyzes were performed using the statistical program IBM SPSS Statistics 23 (Armonk, NY: IBM Corp). The normality of the variables was tested using the Kolmogorov-Smirnov test. To describe the study variables, measures of central tendency (median) and dispersion measures (interquartile range) were used for continuous variables, and absolute and percentage values for categorical variables, with Pearson’s Chi-square test between sociodemographic variables and gender. When the expected values in the table cells were less than five or when the sum of the column value was less than twenty, Fisher’s exact test was used. Mann-Whitney U test (nonparametric variables) was used to analyze the association of quantitative and qualitative variables. When the qualitative variable had three or more categories, the comparison between the means was performed by the Kruskal-Wallis test (using the Mann-Whitney U Test two by two to identify the differences). To identify the correlations between the two dependent variables, Spearman’s correlation test was used. The Receptor Operation Characteristic Curve for MetS to determine TyG index was created ( 12 - 14 ), and the cutoff point for IR was elucidated by two methods: the area under the curve (AUC) that had the best sensitivity and specificity values for the test in question, and the Youden Index [(sensitivity + specificity) – 1]. The level of significance adopted was α < 5%.

This article is derived from the project financed by the Research Program of the Unified Health System (PPSUS), through the notice Fapes/CNPq/Decit-SCTIE-MS/SESA nº 05/2015 – PPSUS, entitled “Health conditions and associated factors: a study on farmers in Espírito Santo”. The study was approved by the Research Ethics Committee of the Health Sciences Center of the Federal University of Espírito Santo (UFES), under number 1,856,331 (CAAE 52839116.3.0000.5060). All participants signed the Free and Informed Consent Form (ICF).

## RESULTS

Table 1 describes the sociodemographic characteristics of the sample, according to gender. In the sample of 790 family farmers, 52.3% (n = 413) were men. In the sample, there was a predominance of individuals in the age group from 31 to 40 years old (n = 231; 29.2%); married or living in a stable relationship (n = 698; 85.5%); with less than four years of study (n = 533; 67.5%); who self-reported white ethnicity (n = 702; 88.9%) and socioeconomic class C (n = 395; 50.0%).

**Table 1:** Sociodemographic characterization of family farmers from a Brazilian region, by sex

Variables		Sex		p-valor	Total n (%)
		Male n (%)	Female n (%)		
Age (decades)				0.655	
	≤30	106 (25.7)	107 (28.4)		213 (27.0)
	31-40	122 (29.5)	109 (28.9)		231 (29.2)
	41-50	100 (24.2)	95 (25.2)		195 (24.7)
	>50	85 (20.6)	66 (17.5)		151 (19.1)
Marital status				**<0.001**	
	Single	48 (11.6)	11 (2.9)		59 (7.5)
	Married or living in a stable relationship	345 (83.5)	333 (88.3)		698 (85.8)
	Separated, divorced or widowed	20 (4.8)	33 (8.8)		53 (6.7)
Schooling (years)				0.620	
	<4	273 (66.1)	260 (69.0)		533 (67.5)
	4-8	96 (23.2)	77 (20.4)		173 (21.9)
	>8	44 (10.7)	40 (10.6)		84 (10.6)
Ethnicity				0.308 *	
	White	362 (87.7)	340 (90.2)		702 (88.9)
	Nonwhite	51 (12.3)	37 (9.8)		88 (11.1)
Socioeconomic class				**<0.001**	
	A/B	42 (10.2)	16 (4.2)		58 (7.3)
	C	222 (53.8)	173 (45.9)		395 (50.0)
	D/E	149 (36.1)	188 (49.9)		337 (42.7)

Chi-square test.

* Fisher’s exact test.

In bold: statistically significant values (p < 0.05). N = 790.

The mean TyG index values in the diagnosis of MetS are described in Table 2 . It is observed that the median TyG index is significantly higher among individuals diagnosed with MetS in both diagnostic criteria (p < 0.001).

**Table 2:** Median values of the TyG index according to the diagnostic of metabolic syndrome in the population of family farmers in Santa Maria de Jetibá/ES

Metabolic syndrome		TyG index		
		Median	IIQ	p-value
NCEP Criteria	Absent	4.39	4.25-4.52	**<0.001**
	Present	4.81	4.59-5.00	
IDF Criteria	Absent	4.38	4.25-4.51	**<0.001**
	Present	4.79	4.57-4.97	

Mann-Whitney U test. Statistically significant values (p < 0.05). IIQ: interquartile range; NCEP: National Cholesterol Education Program’s Adult Treatment Panel III; IDF: International Diabetes Federation.

Table 3 shows the values of the TyG index according to the number of MetS components. In both diagnostic criteria, the average TyG index value increases as the components of MetS are aggregated, reaching Ln 5.18. There was a statistical difference between all categories, with the exception of “4 components” with “5 components”, for both criteria.

**Table 3:** Values of the TyG index according to the aggregation of the MetS components of family farmers in Santa Maria de Jetibá/ES

Metabolic syndrome		TyG index		
		Median	IIQ	p-value
NCEP Criteria	None	4.31	4.21-4.45	**<0.001**
	1 component	4.38	4.25-4.50	
	2 components	4.54	4.40-4.69	
	3 components	4.69	4.53-4.86	
	4 components *	4.96	4.87-5.23	
	5 components *	5.18	5.10-5.19	
IDF Criteria	None	4.30	4.20-4.45	**<0.001**
	1 component	4.35	4.23-4.47	
	2 components	4.47	4.37-4.63	
	3 components	4.69	4.53-4.82	
	4 components *	4.96	4.83-5.16	
	5 components *	5.18	5.10-5.19	

Mann-Whitney and Kruskal-Wallis tests, using Mann Whitney U test two by two to identify the differences. Statistically significant values (p < 0.05).

* Mann Whitney U test two by two identified differences between all tested categories, with the exception of 4 with 5 components of MS, in both diagnostic criteria.

IIQ: Interquartile range; NCEP: National Cholesterol Education Program’s Adult Treatment Panel III; IDF: International Diabetes Federation.

Table 4 describes correlations between the components of MetS with TyG index ( Table 4 ). The correlations between SBP (ρ = 0.258), DBP (ρ = 0.258) FPG (ρ = 0.387) and HDL-c (ρ = -0.351) with TyG index were weak, while WC (ρ = 0.468) had a moderate correlation, and TG (ρ = 0.958) correlated strongly with TyG index. It is important to highlight that the correlations between TyG index with FPG and triglycerides were expected, since these variables are used to calculate the TyG index.

**Table 4:** Correlations between the TyG index and the components of MetS in family farmers in Santa Maria de Jetibá/ES

Metabolic syndrome	TyG index	p-value
Waist circumference	0.468	**<0.001**
Systolic blood pressure	0.258	**<0.001**
Diastolic blood pressure	0.285	**<0.001**
HDL-c	-0.351	**<0.001**
Fasting plasma glucose	0.387	<0.001*
Triglycerides	0.958	<0.001*

Spearman correlation. In bold: statistically significant values (p < 0.05).

* Expected values, since these variables are used in the calculation of the TyG index. HDL-c: high density lipoprotein.

**Figure 1:**
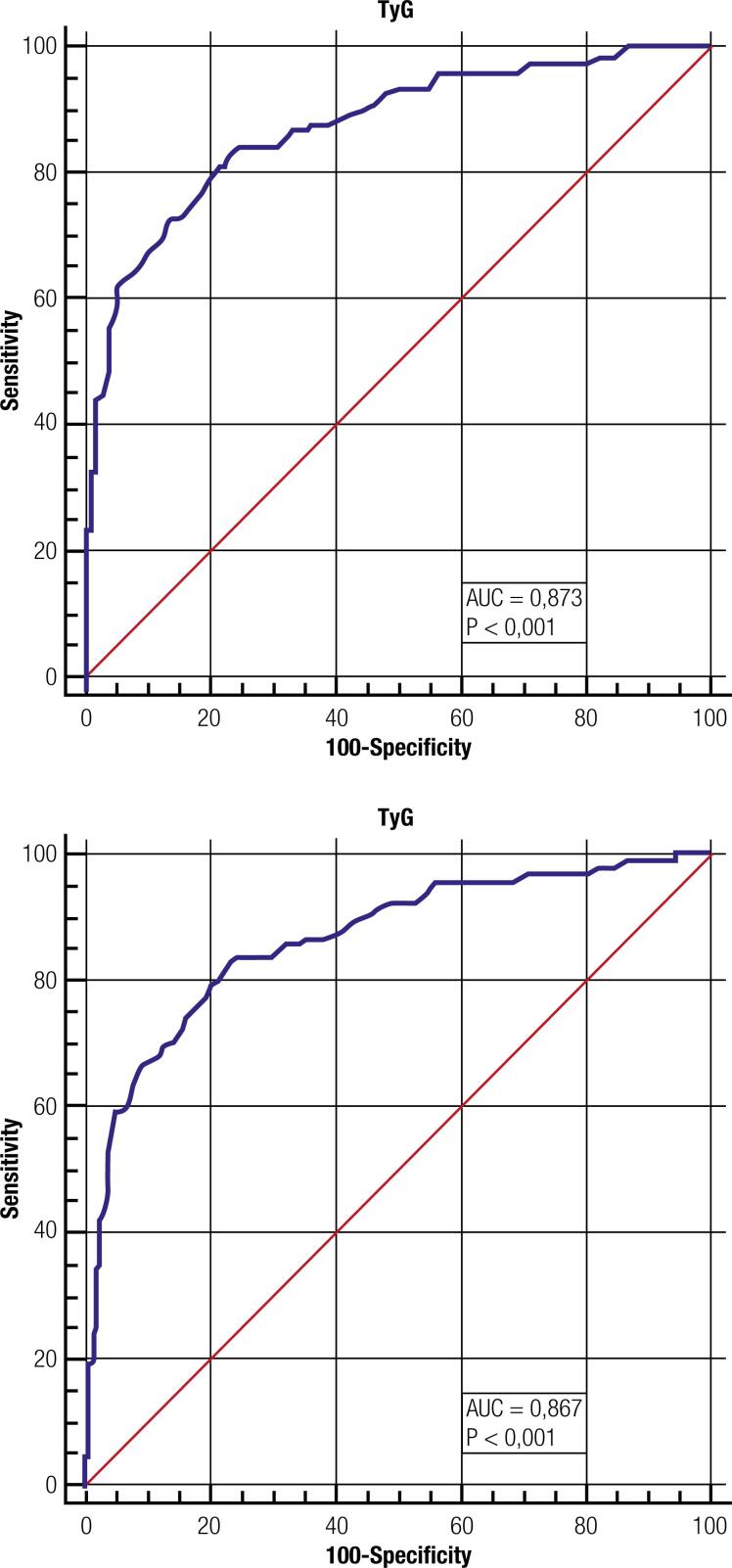
ROC curves of TyG index for metabolic syndrome according to NCEP and IDF criteria, respectively, for rural population in Brazil.

The ROC curves of the TyG index for MetS in the definitions of NCEP and IDF are shown in Figure 1 . The area under the curve (AUC) according to NCEP revealed values of 0.873 (0.848-0.896; p < 0.001). The cutoff point according to the Youden index was Ln 4.52, with sensitivity and specificity of 84.30% and 75.75%, respectively, and a positive likelihood ratio of 3.48 and negative likelihood ratio of 0.21. For the IDF criteria, the AUC was 0.867 (0.842-0.890; p < 0.001), with a Youden cutoff point of Ln 4.55 (sensitivity 80.0% and specificity 79.82%), with a positive likelihood ratio of 3.96 and negative likelihood ratio of 0.25. In order to stipulate a single cutoff point for both MetS diagnostic criteria, Ln 4.52 was defined, since also in the IDF criteria it still presents good sensitivity (82.96%) and specificity (76.76%).

## DISCUSSION

The present study demonstrated that the TyG index is a simple and reliable tool to detect IR in a population of rural workers. Median TyG values were higher in both MetS diagnostic criteria, and increased as MetS components were added. A cutoff point for the TyG index was also proposed for adults living in rural areas of Brazil. This cutoff point for IR can be adopted both in epidemiological studies and clinical practice.

Simental-Mendía and cols. (2008) ( 3 ) initially proposed the TyG index as a simple and low-cost alternative for identifying insulin-resistant individuals, and found similar behavior between TyG index and HOMA-IR values according to the participants’ metabolic glucose status. Guerrero-Romero and cols. (2010) ( 10 ) concluded, based on their study in a population with and without changes in glucose metabolism, that TyG index had good sensitivity (96.5%) and specificity (85.0%) when diagnosing IR, when compared to HIEC. In Brazil, TyG index was validated by Vasques and cols. (2011) ( 11 ) as a viable substitute for the gold standard in the detection of IR, showing better correlations with various parameters of adiposity, metabolic and subclinical atherosclerosis related to IR when compared to HOMA-IR.

A recent study of systematic review and meta-analysis assessed the diagnostic capacity of the TyG index by comparing studies around the world. Diagnostic accuracy varied according to the reference standard and the definition used to identify IR. Studies that used the HOMA-IR as a reference standard showed lower diagnostic accuracy measures in general when compared to studies using HIEC. The authors of this review do not deny the relationship between IR and the TyG index. However, they highlight inconsistent results of the TyG index’s ability to discriminate between individuals with and without IR. In addition, the lack of a standardized definition of RI could limit its clinical usefulness ( 31 ).

In this study, the median TyG index values were significantly higher among individuals diagnosed with MetS when compared to those without MetS. In addition, the median of TyG index also increased according to the aggregation of the MetS components, and a good correlation was found between TyG index and the components of MetS alone, as was also verified by Unger and cols. (2014) ( 8 ).

Abdominal obesity, assessed through the WC, is one of the fundamental components of MetS, and increased values of this marker reflect an increased risk of cardiovascular disease. In part, this stems from the observation that ectopic body fat is related to a range of metabolic abnormalities, including decreased glucose tolerance, decreased insulin sensitivity and adverse lipid profile ( 32 ). This set of abnormalities is called glycolipotoxicity and plays a key role in the modulation of IR.

In obesity, the inflow of lipids can exceed the storage capacity of adipose tissue and result in the accumulation of lipids in ectopic sites, such as the liver and muscle ( 33 ), and consequently has a large proportion of fatty acids entering the mitochondria ( 34 ). The accumulation of triglycerides in the muscle and liver due to insufficient mitochondrial oxidation of fatty acids has been identified as one of the causal factors in the development of IR ( 35 ), where the action of insulin is prevented by inhibiting binding to its receptor, leading to a decrease in synthesis hepatic glycogen levels and reduced muscle glucose uptake ( 4 ). The competition for oxidation and absorption between glucose and fatty acids results in impaired glucose metabolism by the oxidation of fatty acids ( 35 ). Furthermore, the increase in triglyceride levels in individuals with visceral obesity may be attributable to IR, corroborating the importance that triglycerides have in the pathogenesis of IR and the biological plausibility of using triglycerides as a parameter in the identification of IR ( 3 ).

Elevated values of systolic and diastolic blood pressure and an unfavorable lipid profile, represented by elevated TG levels and reduced HDL-c, are components of MetS and in the present study correlated with TyG index. Incident arterial hypertension is associated with disorders in the metabolism of lipoproteins, especially elevated plasma TG and reduced levels of HDL-c, probably due to endothelial dysfunction ( 16 ). While TG and LDL-c reduce the release of nitric oxide, HDL-c stimulates its production and inhibits the adhesion of monocytes to the endothelium, being associated with a protective effect ( 36 ). Sánchez-Íñigo and cols. (2016) ( 16 ) found in their cohort that the TyG index was independently associated with a risk of incident arterial hypertension, and the authors correlate this finding with the state of glycolipotoxicity mentioned above. In China, Jian and cols. (2017) ( 17 ) also found that a high value of the TyG index was significantly associated with the risk of hypertension.

The accumulation of visceral adipose tissue is associated with increased production of inflammatory cytokines, RI ( 37 ), and also induce vascular endothelial dysfunction and autonomic dysfunction ( 38 ). HDL-c stimulates the production of nitric oxide, inhibits monocyte adhesion to the endothelium and has antioxidant and antithrombotic effects ( 36 ) while LDL-c and TG reduce the release of nitric oxide and, secondarily, cause endothelial dysfunction ( 39 ). Changes in endothelin-131 receptor expression, damage to renal microvasculature ( 40 ), impaired arterial compliance and increased arterial stiffness ( 41 ), or IR and hyperinsulinemia ( 42 , 43 ) may be other mechanisms involved in the pathogenesis of hypertension.

The best cutoff point for the detection of IR in a rural population was Ln 4.52, with good sensitivity and specificity. This value is similar to that found by Simental-Mendía and cols. (2008) ( 3 ) in adult residents of Mexico. However, with the cutoff point of Ln 4.65, the authors did not verify good specificity (45.0%) and the sensitivity value (84.0%) was similar to that found in the present study. Still in this study, the authors comment that lowering the cutoff point to Ln 4.60 increases the sensitivity to 91.3%, which confirms the validity of the test for early detection of IR. Knowledge of the properties of the test as to its accuracy in determining IR is essential for application in epidemiological studies, since it allows correcting estimates of disease prevalence due to classification errors (false positives and false negatives). However, considering that the TyG index is used mainly as a screening test for individuals at risk of developing comorbidities associated with IR, a higher sensitivity value is interesting in its use in clinical practice.

Using HIEC as a reference test, the cutoff values for the TyG index ranged from 4.55 to 5.88 with a sensitivity > 67% and a specificity of 32.5% to 85% in 4 studies with a combined population of 678 participants. AUC was the most consistently reported statistical measure among studies (0.596-0.858) ( 31 ). Against HOMA-IR, cutoff values were reported in 5 studies (4.55-4.78) with sensitivity and specificity values ranging from 73% to 90% and 45% to 99%, respectively. The AUC values ranged from 0.69 to 0.89. Notably, all studies used different cutoff values of the HOMA-IR to define IR, limiting its comparability ( 31 ).

Using the diagnosis of MetS to determine the TyG index cutoff point, several authors found discrepant results. In Argentina, researchers found a cutoff point of Ln 8.80, with sensitivity and specificity of 79% and 86%, respectively ( 8 ). In China, the ideal cutoff point was Ln 4.90, with 82% sensitivity and 86% specificity ( 44 ). Moon and cols. (2018) ( 45 ) found a cutoff point of Ln 4.76 for Korean men and 4.71 for women.

A plausible explanation for such differences is the way in which TyG index is calculated. According to the creators of formula ( 3 ), the calculation of TyG index follows the order: the value of fasting triglycerides is multiplied by the fasting glucose, then divided by two, and finally determines the natural logarithm value. However, in some studies the order of determining the natural logarithm and dividing by two is reversed, thus finding high values of the TyG index.

To date, this is the first article using MetS as a parameter to diagnose IR in Brazil and also the first to identify a TyG index cutoff for the rural population in the country. Considering the difficulties of access and health surveillance faced by residents of rural areas ( 22 ), this index can represent an advance in terms of preventing the consequences generated by IR, since it stands out mainly as a screening tool for IR assessment and potentially for DM2, since it uses simple, low-cost biomarkers often used in clinical practice ( 46 ).

This study has some limitations. First, it was not possible to determine the HOMA-IR or the HIEC to confirm the diagnosis of IR. However, several studies have already compared the methods and validated TyG index as a predictor of IR ( 3 , 10 , 11 ). The study is also limited by its transversal character, which requires greater caution in interpreting the results due to the possibility of reverse causality. In addition, the differences in the calculations of the TyG index formula may represent a limitation, since they make it difficult to compare the data found with those available in the literature.

In conclusion, the TyG index is a reliable marker for identifying insulin-resistant individuals, and correlates with the metabolic changes present in MetS. A cutoff point of Ln 4,52 has good sensitivity and specificity in both diagnostic criteria of MetS, being useful both in clinical practice and in epidemiological studies, and can represent an important tool for the creation of protocols for promotion, protection and even recovery health of rural populations.
